# Quantitative and Qualitative Production of Species *Cucumis metuliferus* and the Potential for Adaptation in the Context of Current Climate Change

**DOI:** 10.3390/plants13131854

**Published:** 2024-07-05

**Authors:** Rodica Soare, Maria Dinu, Cristina Babeanu, Mariana Niculescu, Marin Soare, Mihai Botu

**Affiliations:** 1 Department of Agricultural and Forestry Technology, Faculty of Agronomy, University of Craiova, 19 Libertății Street, 200421 Craiova, Romania; rodica.soare@edu.ucv.ro (R.S.); mariana.niculescu@edu.ucv.ro (M.N.); marin.soare@edu.ucv.ro (M.S.); 2 Department of Horticulture & Food Science, Faculty of Horticulture, University of Craiova, 13 A.I. Cuza Street, 200585 Craiova, Romania; mihai.botu@edu.ucv.ro; 3 Department of Chemistry, Faculty of Sciences, University of Craiova, 13 A.I. Cuza Street, 200478 Craiova, Romania; cristina.babeanu@edu.ucv.ro

**Keywords:** harvesting stage, kiwano, production, quality

## Abstract

*Cucumis metuliferus* E. Meyer ex Naudin belongs to the family *Cucurbitaceae*. It is an annual vegetable species known as horned melon or kiwano. Fruits are recommended for the daily diet because they provide vitamins, minerals, and phytochemicals to alleviate malnutrition and improve human health. In this study, kiwano was cultivated in the climatic conditions of Southwestern Romania, which is characterized by warm summers, lower precipitation, and high temperatures, ensuring optimal conditions for growth and development. The fruits were harvested at three stages of consumption: immature stage (green), intermediate stage (white–yellow) or the mature stage (yellow–orange). They were also subjected to analysis on the productive yield and physical–chemical properties. The results showed that the number of fruits ranged from 13.9 to 15.3 fruits/plant, and their average weight had values from 204 g to 234 g, depending on the harvest stage. The results obtained for bioactive compounds and antioxidant activity indicated significant differences (*p* ≤ 0.05), depending on the harvesting stage. The highest values for the antioxidant capacity (140.90 μM TE/100g DPPH) and total polyphenols (48.78 TPC mg/100 g) were recorded in the immature fruit phase, as well as for the carbohydrate content (3.56%), total protein (1.98%), ascorbic acid (4.3 mg/100 g f.w.), and carotene (0.98 mg/100 g) in the mature fruit phase. These results may stimulate interest in the introduction of this species as a niche crop and for consumption as a source of natural antioxidants for the prevention of diseases due to oxidative stress.

## 1. Introduction

By 2050, it is estimated that the world’s population will exceed 9 billion [1]. This rapid increase influences food resources in the sense that global demand in the period from 2019–2050 will be approximately 50–60% higher [2]. The reduction of arable land due to urbanization, as well as climate change, significantly affects the diversity of species and the production obtained [3]. Access to nutritious and sustainable food, now and in the future, is a primary concern for the alleviation of hunger. Climate change represents a global challenge that humanity is facing today because temperatures are continuously increasing, which triggers numerous extreme weather events, such as heat waves, drought, and floods. All of these have a direct impact on crop yield and quality. Some of them can easily adapt to increasingly variable climatic conditions, which requires more thorough research of less cultivated species that have high adaptive capacity and high production.

Climate change has revealed the crucial need to create new varieties of vegetable species with high resistance to abiotic and biotic stress. Local and traditional varieties, as well as genetic diversity from wild relatives of domestic species, provide rich resources for climate-tolerant vegetable-breeding programs [4].

Currently, cereal or vegetable species are grown in large areas using improved varieties and modern agricultural technologies. However, there are many plant species still underexploited or neglected, which develop in increasingly variable environmental conditions, having high genetic tolerance and important nutritional qualities. These desiderata force researchers to find new valuable food sources that are rich in active compounds and adapted to new growing conditions in the context of climatic pressure.

*Cucumis metuliferus* E. Meyer ex Naudin (kiwano), a species in the family Cucurbitaceae, is especially widespread in tropical and subtropical regions, developing at an altitude from 210 m to 1800 m [5,6]. The natural distribution of this species is found in Sub-Saharan tropical and subtropical regions of Africa, stretching from Senegal to Somalia and South Africa. Kiwano is grown commercially for export to Kenya, New Zealand, France, and Israel [7] due to the presence of essential nutrients, minerals, and organic compounds in the fruit. It has been recognized by the WHO as an essential fruit in the fight against disease and malnutrition [8].

*Cucumis metuliferus* is an annual species that tolerates a wide range of soil types, from less deep or deep sandy soils, to well-drained alluvial [5,9] to clay soils or rocky slopes [8]. It is a climbing plant with a well-developed root system, long stems, leaves arranged alternately, a simple ovate-cordate covered with hairs, and yellow flowers. Kiwano shows rapid growth, indicating that it behaves like a late plant in areas with higher temperatures. During growth, plants can tolerate moderate water stress [10]. However, the crop is little known among farmers and consumers around the world despite its many agronomic, nutritional, medicinal, and economic advantages [11,12]. Most often, the fruit is consumed, and in some parts of Africa, young leaves are also consumed [13]. The exotic fruit is eaten raw in salads, in fish and seafood dishes, or it is used in pharmaceutical preparations due to its valuable biochemical composition. It can be eaten raw, in the immature stage, or in full ripening, in the mature stage. The fruits of the immature stages have the appearance and taste of cucumbers [7], and the mature ones can taste of melon with aromatic notes of banana and lemon. Fruits are of ellipsoidal form, about 12 cm long and 8 cm in diameter with a spiked cortex, which is why it is also called horned melon. Inside, the mesocarp is composed of a slightly mucilaginous green, juicy mass with numerous smooth white seeds rich in various phytochemical components that are important in the daily diet [5,6,14]. The fruit has a high nutritional value and a high economic level but has not been fully exploited [15]. Fruits can be stored at room temperature for up to half a year. They can be frozen or dried but not stored in the refrigerator [16]. The fruit’s growing popularity is due to the abundance of food and health benefits. In Europe and North America, kiwano is currently marketed as a luxury fruit, as it is appreciated for its attractive appearance and long shelf life [6,7].

Kiwano is a fruit that meets various needs with numerous food properties. The pulp is very rich in water and has a high concentration of mineral salts, lipids, and carbohydrates [13]. Including it in the human diet can help maintain healthy cholesterol levels and prevent conditions such as heart attacks and strokes [5,16]. The nutritional value and functional properties, such as the anti-cardiovascular, antidiabetic, antiulcer, antioxidant, anti-inflammatory, antimalarial, and antiviral properties, associated with this species suggest its medicinal and pharmaceutical properties [5]. The fruits contain various concentrations of alkaloids, flavonoids, saponins, tannins, glycosides, terpenoids, and phenols, and they are rich in Vitamins C, E, D, B_9_, B_2_, K, B_1_, and β-carotene, as well as iron, potassium, phosphorus, magnesium, zinc, calcium, copper, and sodium [6]. Therefore, they could be used as a dietary supplement and in various pharmaceutical preparations.

Recent studies highlight the potential of the fresh fruit juices of *Cucumis metuliferus* E. as promising and attractive sources of valuable phytochemicals and natural antioxidants for the development of health products and medicines [13,17]. They also discuss the potential economic capitalization of the species as a source of bioactive compounds for functional and nutraceutical foods, as well as for the active food-packaging-production sector to extend the shelf life of packaged foods [5]. Kiwano is grown mainly in conventional systems, but its resistance to attacks of diseases and pests recommend it also for organic culture. Fruits can be harvested in a staggered order, thus ensuring continuous availability and access to food, which is an important dimension of food security. African horned cucumber fruits are nutrient-rich when the species is grown at moderate water stress, on clay or sandy soil, in the open field, or under a shade net [18]. However, it is not known whether the harvest stage influences the nutrient concentration in kiwano fruits since the species does not overcome the barrier of cultivation and use as a niche plant, due to cultural barriers and insufficient knowledge of the productive potential in the conditions of climate change. This desideratum led to the establishment of the objective of this study, that of determining the productive and nutritional potential of the species, on three stages of harvesting (immature green, intermediate, and ripe), as well as its adaptability in the climatic conditions of Southwestern Romania.

## 2. Results and Discussions

The results of the present study refer to the main morphological and nutritional characteristics of kiwano fruits harvested at three stages of consumption (green fruit stage, intermediate, and complete ripening).

### 2.1. Morphological Features of Fruit

Kiwano’s morphological features are shown in Table 1 and refer to the average weight of a fruit, the number of fruits per plant, and the fruit yield per plant.

The number of fruits per plant ranged from 13.9 (mature stage) to 15.3 (immature stage), meaning there was a slight decrease in the number of fruits as they ripened. In the literature, there is a great variability in the number of fruits per plant, which is influenced by the genotype, culture area, and environmental conditions. In a study conducted by [19], in various agro-ecological areas in the eastern, central, and western regions of Kenya on 19 accessions of kiwano, the number of fruits ranged from 7.33 to 16.5, depending on the genotype and crop season. Also, different weed-management practices and sowing times can be significantly affected by the kiwano yield and number of fruits per plant [20].

When measuring fruit weight, we recorded an average variation from 204 g in the immature green fruit stage to 234 g in the mature stage. It can be said that as the fruit matures and breathing intensifies, its weight increases. Currently, reports have found a high variability in the weight of Kiwano fruit, as it has ranged from 194.67 g to 259.33 g [19], or from 104.1 g to 722.2 g, depending on the season, soil type, and culture system [18].

Fruit yield per plant (kg) recorded higher values in the green fruit phase of 3.12 kg/pl, and in the mature stage, it was 3.02 kg/plant. This character correlates positively with the number of fruits and the average weight of the fruit per plant (Table 1). In a study conducted by [21] on kiwano production in response to nitrogen fertilization, at a dose of 154.44 kg ha^−1^ N, kiwano had a reported average number of 2.18 fruits/plant, with an average weight of 237.07 g/fruit and estimated production at 516.41 g/plant.

### 2.2. Degree of Pulp Firmness

The degree of pulp firmness is an indicator of the fruit’s quality; it is the final index by which consumers decide to buy. Changes in firmness influence the time of purchase and the time of harvest. In the present study, the pulp firmness was at an immature stage (green) of 1.55 kg/cm^2^, reaching the value of 1.29 kg/cm^2^ at the mature stage (orange). It is established that at the stage of maturity, the fruits are slightly softened relative to those harvested as immature fruits. During the period of ripe fruits, a slight loss of firmness was observed, while there has been a significant increase in their coloration in orange. During the ripening of fruits, pulp firmness decreases due to enzymatic degradation of the cell walls. In general, changes in textural attributes can be significantly influenced by the harvest stage and ripening period. Our findings are supported by other authors. In addition, Ref. [22] stated that the firmness attributes (peel, fruit, and pulp) of early-harvested mango fruit do not differ significantly from mid-harvested mango, while the firmness of late-harvested mango pulp is significantly lower than during early- or mid-harvest.

### 2.3. Nutritional Quality of Fruits

The taste of kiwano fruit is influenced by the sugar content expressed by total soluble solids (TSS), but also by acidity. In this study, changes in the TSS content in kiwano fruits varied depending on the harvest stage. Thus, at the stage of immature fruit, the content in TSS was 4.2% Brix, and then it increased to a concentration of 5.5% Brix in the mature-fruit stage (Table 2). The concentration of nutrients in fruits is influenced by the varied culture environments, the culture area, or the maturity stage [10,23]. Thus, in the fruits of the kiwano grown under conditions of moderate water stress, both in the shade environment and in the open field, the total soluble sugars in the fruits varied between 8 and 16 Brix [10]. In the subtropical climatic conditions of the Cantareira region, Sao Paulo, the content in TSS of the kiwano fruits was 3.03% Brix [24], and in conditions of the temperate climate in the North Macedonia, Kochani region, in a study conducted by [25], a total soluble substance content of 4.07% Brix was reported. According to [26], TSS ranged from 4.02% in mature green fruits to 6.19% in mature yellow–orange fruits.

Therefore, a relatively high level of total soluble sugar in fruits is important for nutrition and processing, especially when the Brix level is above 5. The values obtained in this study are higher at the stage of maturation (yellow fruit), which recommends it for fresh consumption and juice. Sugar is considered to be one of the basic taste qualities for most fruits and vegetables. Sweet taste induces consumer preference.

In this study, the value of reducing sugar and glucose content was higher in the mature fruit stage. Thus, increasing values of sugar from immature fruits to mature fruits, (2.80% to 3.56%) and glucose (1.46% to 1.94%) were recorded. There are significant differences (*p* ≤ 5) between the three stages of fruit harvesting (Table 2). The results obtained are consistent with those reported by other authors. Thus, Ref. [27], in their study of mango fruits grown in Kenya and harvested at three harvest stages (unripe, inter, and ripe), reported that the three sugars tended to increase with fruit ripening. The increase in reducing sugars in the full ripening phase is due to the hydrolysis of starch and sucrose into simple sugars in the baking processes.

Total soluble sugars from fruits are involved in regulating metabolism and maintaining the body’s energy balance. The sugar content can also be influenced by other factors, such as genotype, culture system, and cultivation area. The conclusions of this study demonstrated that the time of harvest affects soluble sugars in kiwano fruits harvested at different stages. Ref. [19] reported a sugar content variable according to the culture season and genotype, in the first season, between 2.14 and 4.63 g/100 g, but increased in the second season by 3.07 to 5.09 g/100 g. Sugar levels in kiwano are relatively low compared to other fruits in the family *Cucurbitaceae*; 3.73% were reported in reducing sugar [28] or 845.23 mg/g total sugar [23]. The climatic conditions, namely the high thermal values, as well as the low relative air humidity, during the 3 months of fruiting (June–September), which favored a good accumulation of sugar content for the kiwano crop established in the field. This statement is also supported by [18], who found that the culture established in the field on clayey soil, and high-water stress had higher values compared to the culture established in protected spaces or a culture protected with a shading net.

In this study, the acidity values fluctuated depending on the harvest phase. Thus, the acidity decreased from 0.83% in the immature stage to 0.75% in the intermediate stage, and in the mature fruit stage, the values increased. This decrease may be due to the use of organic acids in respiration to provide the energy needed for other syntheses, especially fruit-specific ripening substances [29]. Similar results have been reported by [27], who found that the maturity stage had a great influence on the total titratable acidity content in mango fruits harvested at different stages. A decrease in acidity during ripening was also found for cucumber fruits [29].

Crude proteins are important for the functioning of the human body because they are essential in the processes through which food is transformed into energy, and they offer benefits in the formation of cells or in the development of the immune system. Protein content was found to be lower in immature-stage fruits and higher in mature-stage fruits, the recorded values being from 1.63% to 1.98%. For this study, variability is due to the accumulation potential on the harvest stage. Some authors have reported higher protein content in kiwano seeds. Thus, Ref. [30] reported a higher protein content in seeds than in pulp of 2.63 g/100 g, and Ref. [31] reported values of 23.2 g/100 g.

Kiwano pulp contains important amounts of carotene. The analyses performed indicated different values, depending on the time of harvest, which ranged from 0.68 mg/100 g f.w. (immature stage) to 0.92 mg/100 g f.w. (mature stage) (Table 2). Vitamin A is essential for vision, and β-carotene (a carotenoid and precursor of biologically active molecules of Vitamin A, such as the retina) is an effective antioxidant for the human body [30]. Also, [10] found significant differences in carotene in African-horned cucumber fruits (kiwano) caused by crop conditions, reporting a variability of 1.5 and 1.7 mg 100 g^−1^ d.w.

The quality of the fruit can be influenced by many factors, such as the age of establishment, environmental conditions, applied technology, or the time of harvest. Most vegetables from Cucurbitaceae have a rich chemical composition, which improves the health and stability of food due to their antioxidant power [17]. The values regarding the antioxidant activity of kiwano fruits at different harvest stages are presented in Table 3.

Ascorbic acid is among the most important nutritional-quality indicators in many horticultural crops and has numerous biological activities in the human body [32]. This vitamin is an important component in human nutrition. It is necessary for the absorption of iron for the health of bones, blood vessels, and skin, helps fight infections, prevents scurvy, and protects the body against oxidative stress associated with the onset of diseases [33].

The ascorbic acid content identified in kiwano was close in value for the two harvesting phases: the immature stage and intermediate stage, with values of 3.1 mg/100 g f.w. and 3.7 mg/100/g f.w., respectively, being recorded, while in the ripening phase, an accumulation of 4.3 mg/100 g f.w. was recorded (significant differences, *p* < 0.05) (Table 3). Determining the optimal time to harvest fruits that increase the Vitamin C content of kiwano could increase its consumption. In this context, mature-stage fruits appear to be more beneficial than immature-stage fruits in the accumulation of Vitamin C. There are different accumulations in the content of Vitamin C in the pulp of kiwano fruits during ripening. Similarly, a considerably higher Vitamin C content during ripening was recorded in citrus reticulata fruits [3]. A higher content of Vitamin C was reported in mature peppers compared to immature ones [2]. Consequently, fruits harvested at immature and mature stages by kiwano may be more beneficial than immature ones. According to [5], the pulp has the highest concentrations of Vitamin C (5.30 mg/g^−1^) compared to the peel and seeds, and [34] reported a higher accumulation in the epicarp (352.48 mg/100 g) than in the endocarp or mesocarp.

Total phenol content ranged from 29.62 to 48.78 mg GAE/100 g in kiwano fruits. By harvest stages, there are significant differences (*p* < 0.05) in the total phenol content, the lower value being in the mature fruit stage (Table 3) compared to the immature fruit stage where the values are higher. Therefore, the total phenol content decreased significantly with increasing maturity. The results obtained in this study are consistent with those reported by [35], who recorded 14.424 mg/L, or [17], who recorded 18.97 mg GAE/mL from the fresh juice of *Cucumis metuliferus*. Concentrations in total phenols can also be influenced by the culture system. Thus, values between 3.1 and 5.8 GAE g^−1^ d.w. were reported in a greenhouse environment, and in the open field, the total phenol content varied between 3.1 and 6.4 GAE g^−1^ d.w. [10].

Ref. [36] prepared different extracts from kiwano fruit powder using three solvent systems: water, ethanol, and 50% ethanol–water (*v*/*v*), and they evaluated the antioxidant and reducing activity (TPC, FRAP, and ABTS). The lowest value of total phenolic compounds was reported for the fruit ethanolic extract of 47.2 mg GAE/100 g^−1^ d.w., while aqueous and hydroalcoholic extracts showed higher values of 89.0 and 101.5 mg GAE/100 g^−1^ d.w., respectively.

Phenolic is recommended in human nutrition for its antioxidant properties, which prevents free radicals from reacting with other molecules in the body and prevents DNA damage. Therefore, the values obtained in this study can contribute to supporting the promotion of the culture of this species for commercialization.

Significant differences in total phenolic compounds were also identified in other fruits harvested at different stages. Some authors have shown that immature harvested fruits have a higher content in total phenols compared to those harvested late, after ripening, due to different metabolites released by plants at different stages of growth in green nuts [37,38], black currant leaves [39], or mango fruit [40].

The antioxidant activities to scavenge 2,2-diphenyl-1-picrylhydrazyl DPPH and 2,2′-azino-bis (3-ethylbenzthiazoline-6-sulfonic acid) (ABTS) radicals provide different results depending on the harvesting stage, with the values being presented in Table 3.

When harvesting fruits, antioxidant activity by the DPPH method both by TE and AsA showed the strongest antioxidant response, while the ABTS method recorded a lower antioxidant response. Regarding harvest time, higher values were recorded in the fruit harvested at the immature stage. Thus, the antioxidant activity by DPPH recorded values from 93.8 μM TE/100 g (fruit harvested at the mature stage) to 140.9 μM TE/100 g (fruit harvested at the immature stage), and from 71.78 μM AsA/100 g (fruit harvested at the mature stage) to 103.0 μM AsA/100 g (fruit harvested at the immature stage).

In respect to the antioxidant activity using the ABTS method, values ranged from 85.59 μM TE/100 g (fruit harvested at the mature stage) to 126.89 μM TE/100 g (fruit harvested at the immature stage) and, respectively, from 17.46 μM AsA/100 g (immature fruit) to 90.80 μM AsA/100 g (fruit harvested at the intermediate stage). Analyzing the values from the point of view of the maturation stage, both for DDPH and for ABTS, the results indicate a high antioxidant effect in the immature fruit phase. These results are in line with the studies reported by [40]. In mango fruits, the DDPH radical was higher during the early stage of fruit development and lower at their maturity.

For example, Ref. [35] reported in *Cucumis metuliferus* the highest content of antioxidant activity of 11.2%, as assessed by the DPPH test, compared to other species in the same family of cucumber (*Cucumis sativus*), bitter melon (*Momordica charantia*), and zucchini (*Cucurbita pepo*). According to some authors, “cocktails” selected from juices of *Cucumis metuliferus* and *T. cucumerina* have increased the antioxidant effect in a synergistic way, which can help the population treat various chronic diseases caused by oxidative stress [17]. The methanolic extract from the bark of *Cucumis metuliferus* has pharmacological properties such as antioxidant, anti-inflammatory, spasmolytic, and antiviral activities [13].

All these results indicate that biosynthesis and the accumulation of biochemical compounds are influenced by several horticultural factors: harvest time, genotype, culture conditions, and extraction method.

The findings of the present study serve as a reference point regarding the nutritional potential of kiwano fruits depending on the time of harvest. Other researchers, such as [18], reported that the growth environment is able to cause variations in the nutrient content of crops.

**Figure 1:**
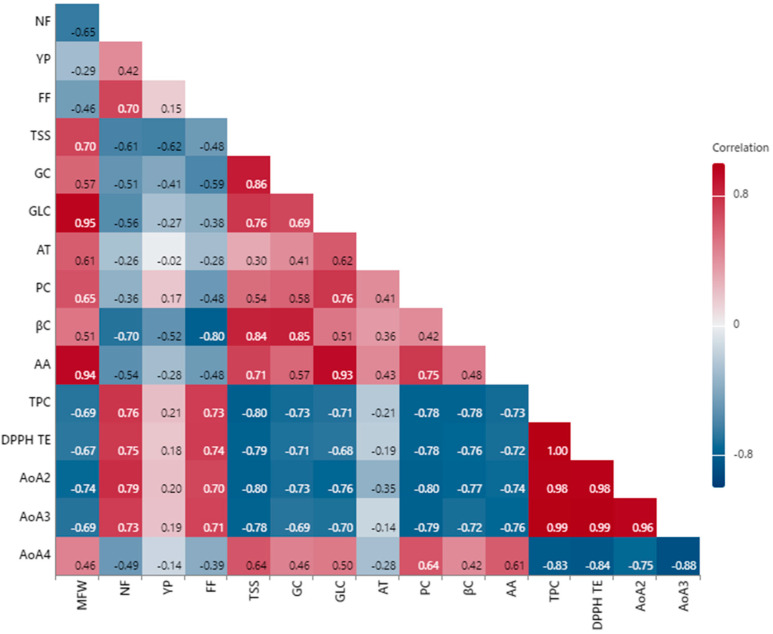
Correlogram between the analyzed parameters (NF—number of fruits per plant; FF—fruit firmness, MFW—mean fruit weight; YP—yield per plant; TSS—Total soluble solids; GC—glucides content; GLC—glucose content; FC—fructose content; AT—titratable acidity; PC—protein content; βC—β-carotene, AA—Ascorbic acid; TPC—total phenolic compounds; AoA1—DPPH TE antioxidant activity; AoA2—DPPH AsA antioxidant activity, AoA3—ABTS TE antioxidant activity, AoA4—ABTS AsA antioxidant activity).

In order to highlight the fruit importance of kiwano fruits, correlations were made between production characters and nutritional parameters. The correlogram shows significant positive correlations between fruit weight and content in glucose and ascorbic acid; between total soluble substance and content in carotene and carbohydrates; between glucose and ascorbic acid; and between the content of total polyphenols and antioxidant activity determined by DPPH and ABTS (Figure 1).

The relatively high correlation of coefficients between phenolic compounds and antioxidant activity values was also reported in the fruits of *Citrus reticulata* [3]. The observed correlations confirm the connection between these characters in the fruits of *Phoenix dactylifera* L. as well [41]. Differences in antioxidant activity could be the result of disparities between harvesting stages and climatic conditions in the growing area, especially variations in temperature and soil moisture, as well as the availability of mineral nutrients in the soil.

Through the quantitative and qualitative yields obtained, the studied species present good adaptability and can be cultivated successfully in the pedoclimatic conditions of Southwestern Romania, which is supported by other researchers who affirmed the adaptability of the species in other areas of the country. Thus, [42] demonstrated the rapid adaptability of the species to environmental conditions for the agroecological zone in the western part of Romania, especially in protected culture but also directly in the field. By expanding into cultivation, this tropical species could have a great commercial potential, and in Romania, as intended for the consumption of certain markets, it could be an excellent source of income for small farmers [43].

## 3. Materials and Methods

### 3.1. Plant Material

The trial was placed in 2018 and 2019 in the experimental fields of the Faculties of Horticulture and Agriculture, University of Craiova, in the Banu Mărăcine Didactical Station (44°18′29″ N, 23°51′43″ E) Romania. Kiwano culture was established through planted seedlings, produced in warm greenhouse at temperature of 20–22 °C. The seeds used in this study are from “Tempus” cultivar and were purchased from the Vegetable Research and Development Station (SCDL), located in Muntenia Region, Romania (Southeastern Europe, 45°09′30.1″ N 26°49′39.2″ E). Planting of the seedling was carried out in the field in the beginning of May for both years of research, in randomized blocks, in three replicates with 12 plants per replicate, in strips of two rows at 60 cm between rows and 40 cm between plants in a row, and the distance between strips of 120 cm. The trial was established on a reddish brown preluvosoil with the following chemical properties: 26.2 mg·kg^−1^ total N, 781 mg·kg^−1^ total P, 334 mg·kg^−1^, total K, 2.67% humus, and pH 6.8. During the vegetation, the plants were trellised and the technology of specific cucumber culture was applied. When planting, the soil fertilization was carried out with 20 t compost to improve soil fertility, and three foliar fertilizers with organic product Cropmax (HOLLAND FARMING B.V, Wezep, The Netherlands) 1 L/ha were applied during vegetation.

### 3.2. Climate Conditions

Weather conditions (air temperature, precipitation level, and relative air humidity) determine the harvest time in cultivated species. Understanding climate variability at the local level helps formulate adaptive responses for crop species in order to manage the risks associated with climate change. The current study took place in the southwest of Romania (44°18′29″ N, 23°51′43″ E) at an altitude of 153 m in temperate climate conditions, with the characteristics of the 2 years of culture presented in Table 4.

The variability of the climate in the southwest of Romania, where the summers are dry and hot, offers favorable conditions for the cultivation of the species *Cucumis metuliferus*. This species needs, for flowering, thermal values between 21–35 °C. It also requires high temperatures for seed germination, plant growth, and fruit development. They are sun-loving plants that require full exposure of at least 6–8 h of direct sunlight per day. *Cucumis metuliferus* grows well in well-drained soils with a slightly acidic-to-neutral pH ranging from 6.0 to 7.5. The soil must be rich in organic matter to promote healthy plant development. Adequate and consistent watering is crucial to the successful cultivation of this species, but it does not tolerate excess moisture. Drip irrigation is recommended to avoid wetting the leaves, thus reducing the risk of fungal diseases [44]. These requirements, of the species in relation to the vegetation factors, are met by the southwestern area of Romania (Table 4). In some months (August and September), it was necessary to apply measures to reduce the effects of the lack of precipitation, which would have led to quantitative and qualitative improvement in fruit yield. In some months (August and September), it was necessary to apply measures to reduce the effects of the lack of precipitation, which would have led to quantitative and qualitative improvement in fruit yield.

**Table 4:** The climatic conditions of the area of Southwest Romania *.

Month	Temperature (°C)						The Relative Humidity of the Air (%)		Rainfall (mm)	
	Minimum		Maximum		Medium					
	2018	2019	2018	2019	2018	2019	2018	2019	2018	2019
May	10.1	5.6	30.0	27.5	19.3	16.1	67	73	57.0	34.0
June	10.0	12.6	32.4	32.2	21.6	22.3	71	75	134.0	140
July	12.6	9.8	31.6	35.6	22.3	22.9	73	66	148.0	64
August	14.8	12.8	33.0	35.5	24.2	25.3	63	51	16.0	8.1
September	1.9	6.7	32.6	32.7	19.2	20.2	61	49	17.0	3.0
October	2.9	3.3	25.7	28.6	14.3	14.3	63	71	5.0	30

* https://rp5.ru/Weather_in_Craiova_(airport) (accessed on 21 June 2024) [45].

### 3.3. Determinations Regarding the Main Production Characters and Fruit Firmness

In order to assess the productive potential, the number of fruits per plant (NF), the mean fruit weight (MFW), and the fruit yield per plant (YP) were determined by repeated weighing, at each harvest, on variants and repetitions, and by harvest stages: immature stage (green), intermediate stage (green–yellow), and mature stage (orange). For this, 15 fruits were harvested (five fruits/repetition) per harvesting stage, and an average sample was made for performing biometric and quality analyses. Fruit firmness (FF) was determined on the central area using a GY-2 fruit penetrometer (Handpi Instruments Co., Ltd., Yueqing, China), which made a point with a diameter of 3.5 mm. The results were expressed as means (in kg/cm^2^).

### 3.4. Determination of Bioactive Compounds

Total soluble solids (TSS) were determined using a digital refractometer (Hanna Instruments Inc., Woonsocket, Rhode Island, 02895, USA) at 20 °C and expressed as a percentage. The titratable acid content (AT) was determined by titration with 0.1 N sodium hydroxide (NaOH) using phenolphthalein as an indicator and expressed as % citric acid [28]. Protein content (PC%) was determined by the Kjeldahl method described by [46]. Nitrogen content in the sample was converted to crude protein content by multiplying the N percentage by the conversion factor 6.25.

The reducing sugars (RS) were extracted in distilled water (1:50 *w*/*v*), 60 min at 60 °C and determined by the colorimetric method at 540 nm with 3,5-dinitrosalicylic acid reagent using glucose as standard [28]. The results were expressed in % fresh weight basis. Glucose (GLC%) content was assayed at 500 nm by glucose oxidase/peroxidase method (GOD/POD), as described by [47]. Glucose oxidase (GOD) is used to oxidize glucose by the oxygen in the air to gluconolactone and hydrogen peroxide. Under the influence of peroxidase (POD), the hydrogen peroxide reacts with color indicator, forming a pink compound. The glucose content was calculated from calibration curve using glucose (5 mg/mL) as standard. β-carotene content was determined according to the method described by [48], with some modification. β-carotene was extracted in 2:1:1 hexane:methanol:acetone (1 g:25 mL) 30 min in the dark. Further, 5 mL of distilled water are added, and the solution is shaked for 5 min. After phase separation, the non-polar layer was collected and spectrophotometrically analyzed versus a blank of hexane solvent. For analyzing the levels of total carotenoids, the absorbance was measured at 450 nm and results were calculated using a value of 2500 for the extinction coefficient (E1%). The results are expressed in mg/100 g f.w. (fresh weight). The content of ascorbic acid (AA) was determined by iodometric method [28]. The extraction from the biological material was done in 2% hydrochloric acid HCl (1:10 *w*/*v*) and filtered. The ascorbic acid was then titrated with 4 mM potassium iodide (KIO_3_) in presence of 1% potassium iodide (KI). The resulting iodide (I_2_) reacts with ascorbic acid, oxidizing it to dehydroascorbic acid. The redox titration endpoint is determined by the first iodine excess that is complexed with starch, resulting in a deep blue–violet color. The results were expressed as mg/100 g f.w [28]. The extracts for the determination of total phenolic content and antioxidant activity were prepared into 80% aqueous methanol (1:20 *w*/*v*) in “Fungilab” ultrasonic bath at 24 °C for 70 min. The resulting slurries were centrifuged at 4000× *g* for 5 min, and the supernatants were collected. The total phenolic content (TPC) was determined colorimetric at 765 nm by Folin–Ciocalteu method [39]. Each extract was mixed with Folin-Ciocalteu’s phenol reagent, Sigma-Aldrich (St. Louis, MO, USA) and saturated sodium carbonate (Na_2_CO_3_) solution. The mixture was allowed to stand at room temperature for 60 min, and then the absorbance was recorded at 765 nm [39]. The total phenolic content (TPC) was calculated using a standard curve prepared using gallic acid and expressed as mg of gallic-acid equivalents (GAE)/100 g f.w. DPPH (2,2-diphenyl-1-picrylhydrazyl) radical scavenging assay: A 0.075 mM DPPH solution in methanol was mixed with sample extracts and vortexed thoroughly. The absorbance of the mixtures was recorded after 20 min at 517 nm, and the percentage of neutralization was calculated [28]. The standard-calibration curves (Trolox-T and ascorbic acid- AsA) were plotted as a function of the percentage of DPPH radical scavenging activity. The final results were expressed as μM TE/100 g f.w. and μM AsA/100 g f.w. ABTS (2,2′-azino-bis (3-ethylbenzothiazoline-6-sulfonic acid) radical cation scavenging activity: ABTS radical cation was produced by reacting 7 mM ABTS stock solution with 2.45 mM potassium persulfate in the dark at room temperature for 12–16 h. Also, 0.1 mL sample extract was mixed with 2.9 mL of ABTS radical cation solution (diluted with methanol to an absorbance of 0.70 at 734 nm). After reaction at room temperature for 6 min, the absorbance at 734 nm was measured, and the percentage of neutralization was calculated [28]. The standard-calibration curves (Trolox and ascorbic acid) were plotted as a function of the percentage of ABTS radical cation scavenging activity. The final results were expressed as μM TE/100 g f.w. and μM AsA/100 g f.w. All the spectrophotometric measurements were conducted with Evolution 600 UV-VIS spectrophotometer, Thermo Scientific (Cambridge, UK), with vision PRO software.

### 3.5. Statistical Analysis

The data obtained were analyzed, and all results were expressed as means. The statistical significance of differences between variants was determined with the analysis of variance (ANOVA). Means that do not share a letter are significantly different according to Fisher LSD method and 95% confidence. Correlogram for the studied characters was also performed.

## 4. Conclusions

The data obtained in this study confirm the significant effect of the harvest stage on productivity and nutrient and antioxidant content. In general, kiwano fruits harvested in the immature stage had a high antioxidant capacity and a high content of total polyphenols. In the mature fruit phase, there was an increased content in total soluble substances, Vitamin C, carbohydrates, total protein, and carotene. Therefore, the optimal harvest time could serve as an indicator for a consumption that targets antioxidant capacity and polyphenols that can block the formation of free radicals in the body or improve the intake of nutrients by a higher content of Vitamin C, carotene, or protein. The productions obtained in this species demonstrate the high capacity of fruiting and adaptation to the environmental conditions of Southwestern Romania. In the period of 5 months, from May to September, there are optimal conditions for fruiting. The increase in temperature values (maximum, minimum, average) determines the extension of the cultivation period in September due to the favorable conditions, and with the decrease in temperature, in October, kiwano ends its vegetative cycle. Kiwano can be a niche crop for farmers, thus increasing crop diversification by using lesser-known species.

## Figures and Tables

**Table 1:** Main average characters of fruit production and firmness.

Harvesting Time	Average Fruit Weight (g)	No. of Fruits/Plant	Yield/Plant (kg)	Fruit Firmness (kg/cm^2^)
V1—fruit harvested at immature stage	204 ^b^	15.3 ^a^	3.12 ^a^	1.55 ^a^
V2—fruit harvested at intermediate stage	212 ^ab^	14.8 ^ab^	3.07 ^a^	1.50 ^a^
V3—fruit harvested at mature stage	234 ^a^	13.9 ^b^	3.02 ^a^	1.29 ^b^

Means followed by different letters are significantly different according to the Fisher LSD method and 95% confidence.

**Table 2:** Nutritional composition of kiwano fruit.

Harvesting Time	TSS (%)	Reducing Sugar (%)	Glucose (%)	Acidity (%)	Protein (%)	Carotene (mg/100 g f.w.)
V1—fruit harvested at immature stage	4.20 ^b^	2.80 ^b^	1.46 ^b^	0.83 ^a^	1.63 ^b^	0.68 ^b^
V2—fruit harvested at intermediate stage	4.90 ^ab^	3.03 ^ab^	1.62 ^ab^	0.75 ^b^	1.82 ^ab^	0.73 ^b^
V3—fruit harvested at mature stage	5.50 ^a^	3.56 ^a^	1.94 ^a^	0.88 ^a^	1.98 ^a^	0.92 ^a^

Means followed by different letters are significantly different according to the Fisher LSD method and 95% confidence.

**Table 3:** Antioxidant activity of kiwano fruits at different stages of harvest.

Harvesting Time	Ascorbic Acid (mg/100 g f.w.)	TPC mg/100 g f.w.	AO DPPH		AO ABTS	
			μM TE/100 g f.w.	μM AsA/100 g f.w.	μM TE/100 g f.w.	μM AsA/100 g f.w.
V1—fruit harvested at immature stage	3.1 ^b^	48.78 ^a^	140.9 ^a^	103.00 ^a^	126.89 ^a^	17.46 ^c^
V2—fruit harvested at intermediate stage	3.7 ^ab^	37.06 ^b^	111.3 ^b^	87.20 ^b^	98.30 ^b^	90.80 ^a^
V3—fruit harvested at mature stage	4.3 ^a^	29.62 ^c^	93.8 ^c^	71.78 ^c^	85.59 ^c^	76.29 ^b^

Means followed by different letters are significantly different according to the Fisher LSD method and 95% confidence.

## Data Availability

Data are contained within the article.
